# Paragons, Mavericks and Innovators—A typology of orthopaedic surgeons' professional identities. A comparative case study of evidence‐based practice

**DOI:** 10.1111/1467-9566.13392

**Published:** 2021-10-27

**Authors:** Amy Grove, Catherine Pope, Graeme Currie, Aileen Clarke

**Affiliations:** ^1^ Warwick Medical School University of Warwick Coventry UK; ^2^ Nuffield Department of Primary Care Health Sciences University of Oxford Oxford UK; ^3^ Warwick Business School University of Warwick Coventry UK

**Keywords:** case study, evidence‐based practice, identity, surgery

## Abstract

Clinical guidelines, as vehicles for evidence‐based practice (EBP) attempt to standardize health‐care practice, reduce variation and increase quality. However, their use for surgery has been contested, and often resisted. This article examines professional responses to EBP in hip replacement surgery using data from case study observations and interviews in three English orthopaedic departments. A professional identity perspective is adopted to explain how standardization through EBP, represents an empirical phenomenon around which surgeons enact their identities as Paragons, Mavericks or Innovators, to enhance legitimacy and stratify themselves in their response to EBP. Attention is drawn to variation between Paragon surgeons working in university (teaching) hospitals and Maverick and Innovator types located in general hospitals, and the ways this interacts with adoption of EBP. The typology shows how practice variation is related to surgeons' tendencies to align to characteristic types, with distinct social processes, power and prestige, and which are in turn influenced by organizational context. The dynamics of EBP and professional identity continues to limit attempts to standardize surgical practice. The typology contributes to the understanding of failures to follow EBP, as associated with the identities individuals create and negotiate, and with identity narratives used to legitimize differing responses to EBP.

**Abbreviations**
ARC: Applied Research Collaboration
EBP: evidence‐based practice
INT: Interview
NIHR: National Institute for Health Care Excellence

## INTRODUCTION

Despite three decades of evidence‐based practice (EBP) and the introduction of clinical guidelines, surgical practice has continued to resist standardization (Briggs, 2015, 2020). Twenty years ago, Pope (2002) demonstrated how contingency could be used to explain surgical variation, and elsewhere studies of surgical practice showed how health‐care professionals legitimized resistance to EBP (Fox, 1992, 1993; McDonald et al., 2006; Tanner & Timmons, 2000; Timmons & Tanner, 2004). The practice of surgery is an enduring topic for medical sociology (Atkinson, 1981; Bosk, 1979; Burkett & Knafl, 1974; Knafl & Burkett, 1975; Swazey & Fox, 1970). Bosk (1979), for example, in his classic ethnography of surgical error, revealed how local standards of surgical performance constitute powerful norms governing surgical communities.

Fox (1992) took up Bosk's arguments in his study of English surgical practice and showed how surgeons legitimize their own practice. Fox sought to ‘identify the key elements in the discourses of surgeons, and of others involved in surgery as a clinical strategy is constituted’ (Fox, 1992: 128) and unpicked the rhetorical repertoires surgeons used to constitute their knowledge and status. He noted that in situations where there is more than one possible specialist technical procedure, the procedure selected will be the one that enhances the prestige and social processes of those individuals with authority. In these ways surgeons achieve power and maintain professional dominance (Fox, 1992; Freidson, 1970; Pope, 2002), using status and position to reinforce professional boundaries and contest definitions of ‘best’ practice proposed by outsiders (Grove et al., 2018, 2020; Powell & Davies, 2012).

In this article, we explore how surgeons continue to resist standardization despite the pervasive growth of surgical guidelines. We take a professional identity perspective to show how surgical variation is generated by surgeons' distinct approaches to practice and how these approaches are constructed to serve and reinforce a specific purpose (Stets & Burke, 2000). Our analysis argues that different approaches to practice cluster around three surgeon identities; Paragons, Mavericks and Innovators. We explore why and how different identities emerge in different organizational settings and show how these identities reveal where and how surgeons source their own sense of legitimacy in relation to EBP, thereby creating surgical variation.

### Standardization in the practice of surgery

Sociological interest in the standardization of surgery was provoked in part by epidemiological studies which revealed persistent practice variation, and the rise of EBP as an attempt to address it (McPherson et al., 1982; Wennberg, 2002). Early studies suggested that variation was attributed to individual differences between specialists, rather than differential distribution of disease (Bloor, Bloor et al.^1976^, ^1978^; Travis, 1985). Berg (1997) later characterized EBP as a set of instructions which attempt to tell clinical professionals how to act, in effect a form of bureaucratic rule (Berg, 1997). Harrison (2002) went on to suggest that EBP reinforced the notion that only research ‘counts’ as valid and reliable evidence and that personal experience was denigrated as a source of valid knowledge.

The standardization of surgery became government policy, yet empirical and theoretical analyses show how health‐care professionals have continued to resist standardization (Mannion & Exworthy, 2017; Timmermans & Angell, 2001; Timmermans & Epstein, 2010). Evidence in the form of protocols and guidelines is changed and re‐appropriated and social relationships, contingent practice and organizational positioning have all been shown to contribute to surgical variation (Berg, 1997; Fox, 1992; Mannion & Exworthy, 2017; May, 2006, 2007; Silverman, 1987; Svensson et al., 2009). Variation has been explained as a consequence of the microlevel action of individuals and the ways that knowledge or evidence become privileged (Delamothe, 1993; Ducey & Nikoo, 2018; Grove et al., 2020; Mannion & Exworthy, 2017; Mykhalovskiy, 2003). McDonald et al. (2006) in particular highlighted how the identities that individuals create and negotiate, and the social norms which correspond to those identities in practice, support resistance to EBP. Drawing from their theoretical approach we use professional identity (Stets & Burke, 2000) to understand how and to what ends (some) surgeons resist standardization.

### Surgical practice and professional identity

In their portrayal of EBP for patient safety, McDonald et al. (2006) found that doctors were resistant to, and managers supportive of, guidelines, but both groups used identity narratives to claim legitimacy and advance group interests. They theorize that professional identity construction is a process by which individuals draw on discursive regimes to provide symbolic resources which can be used for identity negotiations. Subsequently, the negotiations that occur between individuals and groups take place within interdependent but imbalanced power relationships. In this sense, failures to follow standardized practice do not reflect an individual's cognitive problem but instead the identities and positions which individuals generate, inhabit and negotiate in the social world.

The idea that medical professionals distinctly narrativize their work to defend against standardization has explanatory value. However, this tends to underplay variation within and across professional groups, for example, between surgeon–surgeon as opposed to medic–manager. Interprofessional boundaries contribute to practice variation (Grove et al., 2018, 2020; McDonald et al., 2006; Powell & Davies, 2012) but this is only part of the story explaining resistance to standardization. Currie et al. (2012) revealed how professionals seek to stratify themselves from others through identity tactics, noting, in their study of organizational change in cancer genetics services, that doctors self‐identified as ‘innovation champions’ to accomplish this.

This article focuses on one of the most routine and potentially standardizable surgeries performed in the context of orthopaedics; replacement of a patient's hip joint with an artificial implant also known as total hip arthroplasty (Pivec et al., 2012). We draw on these ideas about identify work to show how orthopaedic surgeons describe and understand their practice and practice uncertainty, reinforce their decisions and validate their activities relative to standardization (Bresnen et al., 2019; Sveningsson & Alvesson, 2003). We present a typology of orthopaedic surgeons' professional identities and through abductive analysis, show the ways in which identity work serves a purpose: as a rhetorical device to enhance legitimacy and signify position; as a means for self‐stratification in relation to others; and as a predictor of adherence to EBP.

## STUDY METHODOLOGY

### Methodological rationale

The research grew from a desire to understand why the standardization of practice is stymied in orthopaedic surgery (Briggs, 2015, 2020). Our epistemological positioning is broadly interpretivist. Empirical work was shaped by a preset study protocol (Grove et al., 2015), complemented by which, derived from literature review, we understood surgeon's use of EBP as shaped by their identities (e.g. McDonald et al., 2006). Following this we sought to generate an inductive real‐world representation of surgical practice via the subjective accounts of surgical staff and periods of qualitative observation that revealed the dynamics of EBP and identity (e.g. effects of enactment upon professional legitimacy and stratification in responding to EBP).

### Recruitment and data collection

Data collection was undertaken by AG in the orthopaedic departments of three NHS hospitals in England between 2014 and 2016. During this time a member of the research team was embedded within the hospitals as an observer for a minimum of 3 months. A variety of case study data collection methods were used, including informal and formal interviewing, observations of daily surgical work and documentary analysis. The three departments studied were purposively and pragmatically selected from 240 NHS hospitals providing total hip arthroplasty in 2013 (NJR, 2014), informed by project scoping and initial informal conversations about gaining access. The hospitals' organizational structures varied (at least on the surface) in their approaches to guideline implementation (e.g. no formal process vs. clinically managerially led) (Westert et al., 1993). We selected a designated academic orthopaedic department co‐located in a University (Case A), a nonteaching hospital (Case B) and in a teaching hospital affiliated to a University (Case C).

The Research and Development teams and orthopaedic departmental leads in each case were contacted via email to begin recruitment. These key contacts supplied names of local staff, who were approached directly through a combination of email, telephone calls and in‐person requests. Subsequent interviewees were identified using a snowball technique, following introductions via existing participants. Detailed notes about each case were made by the lead researcher (AG) to illuminate and enhance interview transcripts.

The interviews were semi‐structured around a topic guide derived from themes in the literature on standardization (e.g. Mannion & Exworthy, 2017; Timmermans & Berg, 1997), organizational aspects of surgical practice variation (e.g. Fox, 1992; Pope, 2002; Powell & Davies, 2012) and the release of clinical guidance regarding the conduct of total hip arthroplasty (NICE, 2014). We piloted the topic guide during interviews with two surgeons and amended to improve clarity. Questions were adapted to reflect the individual's profession and role (e.g. departmental administrators and surgical trainees).

During the interviews, we sought to understand views about EBP, and the strategies used in making decisions (e.g. what factors do you consider when deciding to offer surgery to a patient?). Probing explored beliefs about standardization and the relevance of clinical guidance on their practice (e.g. did new guidance change your surgical practice?). This interpretivist narrative approach enabled individuals to expand on topics of interest. Interviews were digitally audio recorded and transcribed by an external professional. Our results draw on the observational field notes collected over 12 months, and interviews with 54 individuals, detailed in Table 1. This combination of interviews and observation allowed credibility checking across the participant accounts, and between participant and researcher accounts.

**TABLE 1:** Role description of interview participants

Role	Number
Consultant‐grade orthopaedic surgeons of mixed subspecialty (arthroplasty, reconstruction, trauma)	15
Orthopaedic surgical trainees	9
Orthopaedic nurses/operating department practitioners	17
Departmental administrators and managers	13
Total	54

The local research ethics committee of each hospital and the host University granted ethical approval for the study. Informed consent was obtained from all participants.

### Data analysis

An abductive thematic approach was used to analyse the data (Braun & Clarke, 2006). After data familiarization, we developed a coding frame for categorizing and organizing the data into relevant first order codes, subcategories and themes (Braun & Clarke, 2006) (see Table 2). This process of identifying, simplifying and ordering the data enabled us to describe the identities in terms which made them comparable and explicable (McKinney, 1969). Transcripts and field notes were explored in team meetings, and to assess agreement. For example, when constructing the typology, we focused on the microlevel differences between surgeons which explored ‘individual beliefs, perceptions and values of orthopaedic practice’.

**TABLE 2:** Typology of orthopaedic surgeons' professional identities: Paragons, Mavericks and Innovators

Example quotes	First‐order codes	Categories	Dimensions of identity	Identity type
‘I always get told I can't do these things (laughs) well, you know, you can look at the difference in my patients’. (INT C 37010) ‘You think they are bad here?…you wait and see some (?… Hospital name) surgeons, absolute mavericks, some of them… implants that are far more expensive than standard hips and knees’. (INT C 377011) ‘I am confident about the decision. It might not be the right decision [but] I’m confident that what we do is the right thing to do’. (INT C 198003)	Variation/special cases, deviant behaviour, beliefs about the profession/orthopaedics are different, confidence to disobey, autonomy, surgeons’ power, competitive, not the bigger picture, works in my hands, my decision to operate	Beliefs about orthopaedic surgery as an exception and special case	Confidence in personal ability to adopt a more nuanced view practice which goes beyond evidence High autonomy and discretion	**Maverick** The ‘showmen/women’ possessing unbounded confidence in their surgical ability. Self‐identified as rebel/ troublemaker. This could bring them into conflict with the norms of their hospital and wider community
‘I came up with an idea to treat patients who have got a wrist fracture. They just didn't get it. It's like … they didn't seem to fathom the concept of it, despite me explaining it and sending them pictures and 3D drawings, they just didn't get it. It wasn't something I could manufacture myself on my kitchen table, like I did with the other one’. (INT C 218007) ‘The innovation…[of different implants] has got to a point, where you're tinkering between the performance of a Maserati versus a Ferrari…you've got to be really confident that you know what you're doing’. (INT C 190004) ‘Obviously most surgeons probably think well I want to go for the latest thing. I just think we've got to always have an eye on the next generation. Otherwise we'll never get beyond … essentially we'll live with the obsolete’. (INT C 218007)	‘Typical patients’ do not need evidence, the way we do it/ have always done, light bulb experience, practice‐based experience, craft and skill versus science, innate passion/enthusiasm, ownership of the process of surgery	Personal experience and innate practice drivers	Internally mandated knowledge‐based jurisdiction High autonomy and discretion	**Innovator** Desire to try new implants and techniques; to improve the orthopaedic field. Visionary approach; innate belief they make a difference to patients and speciality that outweighed the potential (physical, financial and reputational) consequences of their practice
‘The choice of prosthesis [is] essentially the same prosthesis but minor variations in the bearing surface. The evidence base for (brand name) is fairly strong. The evidence base for (brand name) is probably not as strong as it could be’. (INT C 190004) ‘The NHS was ever built to give every patient a (brand name) implant. I think there's too many (brand name) being put in … it's unnecessary. I think a huge number could have been metal and plastic’. (INT C 37011) ‘I don't think it's helpful or innovative for me as a surgeon to be trying something new on my own in the hospital, whereas lots of surgeons would genuinely believe that, I don't think they fully understand what they're doing or the implications of what they're doing and that's because they're not research trained to understand like that, they're trained surgeons, they know how to put the implants in but they may not fully understand the implications of what they're doing and that's the issue. On a very personal level they feel that they're doing something useful for them and the patients but actually probably something really rather unhelpful for everyone’. (INT C 218011)	Intangible decisions, value of legacy knowledge, belief in established treatments, solving problems with science, influence of trainers, strive for personal/professional development, academic credibility	Further training and development and intangible legacy knowledge	Draw on evidence Externally mandated knowledge‐based jurisdiction	**Paragon** Gold standard surgeons who performed the same types of surgery using established implants and techniques. They described their practice as standardized/typical and routine and evidence‐based

As described by Kluge (2000), the result of this systematic grouping process was the identification of a set of character types associated with varying enactment of EBP in orthopaedic surgery. The elements of character types within the groups were similar (they had ‘intern[al] heterogeneity on the level of the type’) and there were strong differences between the character types (‘external heterogeneity on the level of the typology’) (Kluge, 2000). As a check on the credibility of the typology construction, we returned to our field notes and transcripts checking the types against both the subjective accounts of surgeons and our interpretative accounts.

To develop a conceptual understanding of the empirical data and foster our theoretical ideas, we followed a process of abductive analysis (Timmermans & Tavory, 2012).

This abductive approach aims to generate the best prediction, or explanation of a phenomenon using incomplete observations. During our data collection, we identified an empirical phenomenon—the apparent distinction in how surgeons enact their identities—which could not be fully explained by the existing range of identity theories. We explored if these theories fully account for the typology we had constructed (i.e. saturation) or whether more explanatory power could be generated.

Building on what we knew from previous theoretical accounts, alternative explanations emerged as we encountered potentially novel justifications for our empirical phenomena. We set out to choose the ‘best’ explanation among many alternatives in order to explain the purpose of the identity types. This recursive process required double‐fitting of data and theories by revisiting our typology in light of the existing accounts of standardization (Timmermans & Berg, 1997) and professional identity work (Bleakley, 2011; McDonald et al., 2006; Pope, 2003; Powell & Davies, 2012). We made assumptions about our abductive inferences based on the interplay between theories and empirical data, for example, individual codes (e.g. ‘confidence’ ‘uncertainty’) were further explored and refined by rereading and incorporating our theoretical insights and tested and further developed against the subsequent data collected. We underwent a process of defamiliarization to test our theoretical saturation through revisiting our data in different ways, for example, through the use of metaphor, to finally enfold literature (Timmermans & Tavory, 2012).

Through comparative cross‐case analysis we searched for similarities, contrasts and anomalies between the three hospitals, the surgeons and their enactment of EBP drawing on Eisenhardt's (1989) case study road map method to help structure the cross‐case analysis. We moved from our initial data, to within and across‐case pattern searching and identification of divergence. Pushing the analysis further, we explored the impact of ‘place’ and the stability of the typology across differing organizational cultures. We compared within and across cases to search for attributes and meaningful relationships and to sharpen the emerging identity definitions, for example, to understand if ideal types or extreme types were organizationally contingent. Periods of reflection and discussion enabled us to characterize the constructed types and scaffold our themes to our theoretical approaches so that types were categorized along a continuum of practice which mirrored surgeons' propensity to follow EPB—broadly from champion to sceptic.

Finally, we searched the data to look for unexpected findings, inconsistencies and missing data and performed member‐checking with a subgroup of individual surgeons and analytic triangulation via peer‐debriefing (Given, 2008). We considered how in enacting their surgeon ‘type’, role holders were able to legitimize their identity to others within their professional group, and in the process stratify themselves from others in their group. At this final stage, to ensure authenticity of our analysis and assess its local universality, we presented our typology, its antecedents and the effects of its enactment upon professional legitimacy and stratification at seminars attended by a range of staff, including surgeons, at hospital through which we obtained their feedback (Timmermans & Berg, 1997). Readers might note these audiences recognized our depiction of surgeons and effects of its enactment.

## FINDINGS

### The typology of identities in responding to EBP

Variation in surgeons' approaches to practice appeared to be supported by a variety of key factors, including but not limited to learning from one‐off experiences, patterns of practice observed over time, formal and informal training, the social and organizational processes surrounding them and wider cultural and historic forces at play. Whilst constructing the typology, the suggestion that these factors might be associated with distinct professional identities initially came from the in vivo codes offered by our participants. For example, one surgeon was described, and described themself as a ‘complete Maverick’ and in trying to understand what this meant, we began to identify identity types.

We mapped clusters of behaviours, tactics and choices that the surgeons made, and began to identify, order and examine how patterns of practice in response to EBP were generated, reinforced and maintained. This typification process required the pragmatic reduction and equalization of information (McKinney, 1969) so that the identities surgeons inhabit, construct and negotiate, correspond to the typical social and organizational rules to which those identities conform (McDonald et al., 2006), for example, the behaviours and tactics of an ‘ideal Innovator’. The typology was refined resulting in the three types depicted in Table 2: Paragons, Mavericks and Innovators.

The identity types appeared to cohere around three analytic dimensions of identity which were enacted differently by surgeons; the client interest, for example, doing the ‘best’ for the patient; an externally or internally mandated knowledge‐based jurisdiction within which surgeons practice and which differentiates them from others; and finally, surgeons’ autonomy and discretion around decision‐making.

Mavericks might combine client interest focused on their own interpretation of what is the ‘best’ thing to do, with autonomy and discretion around decision‐making to legitimize themselves and their practice (see Table 2). During one professional meeting observation, a Maverick surgeon described how he was able to achieve positive results with a specific implant and procedure because ‘in my hands, I can achieve good results’ (national implant registry data collected from 460 hospitals across the United Kingdom had shown this procedure to be less effective and at times harmful to patients, NJR, 2014) (Observation note). Paragons on the other hand might draw upon client interest grounded on evidence‐based, safe care such that EBP was the externally mandated knowledge‐based jurisdiction within which Paragon surgeons practiced.

Innovators differed as they tended to combine their own internally mandated knowledge base predicated on personal experience with autonomy and discretion to legitimize their work. Innovators labelled knowing what to do as ‘instinct’ or ‘gut feelings’:

“Instinctive reactions can vary in different operations; sometimes things work and sometimes they don't. You don't know until you are ‘in’ there.” (Consultant Surgeon, B)

Such ‘gut feelings’ were marshalled alongside technical knowledge from surgical textbooks and training. The surgeons talked about both: ‘there's the theory of it and then there's the practice of it. So, the theory is well‐established, it's in the textbooks. But the practical aspects of doing a joint replacement…it's different’. (Trainee Surgeon, A). Where Innovators needed to experience how an intervention worked, Mavericks reported wanting to respond to the situation ‘in their hands’. The feel of the surgery was hugely influential for Mavericks and this phrase was frequently mobilized to describe what they did:

“In my hands, that wouldn't be a good choice, changing my practice might actually be more harmful or dangerous than sticking to what I know works.” (Consultant Surgeon, B)

Mavericks used the term ‘in my hands’ in two ways, firstly, to signify the physicality of surgery and secondly, to symbolize their position in the clinical hierarchy achieved through expert practice. The latter was something that only they could achieve and enabled them to signify themselves as different from their colleagues.

Evidence‐based guidance was not the primary source of evidence influencing the surgeons’ practice, but it could be useful for the surgeons in different ways. As noted by Timmermans and Berg (1997), evidence was reappropriated: for Innovators, it was translated into ‘evidence that works for me’. Innovators required experiential evidence:

“When I started doing it, I started testing it on a few patients of my own and when it seemed to be working and I'd got it to a point where it was now becoming useful, I then approached my three or four key colleagues who do most of the hip replacements and said, look, this is what I've done and what do you think and showed them how it worked.” (Consultant Surgeon, B)

A key driver for Innovators was a desire to ‘move the specialty forward, and not stagnate’ and to search for the ‘perfect hip’ replacement (Consultant Surgeon, A):

“I just think we've got to always have an eye on the next generation. Otherwise we'll never get beyond … essentially we'll live with the obsolete.” (Consultant Surgeon, C)

Innovators appeared to intentionally act in a nonevidence‐based fashion like Mavericks. However, they would also adapt EBP, driven by their ambition to ensure that the speciality was not ‘left behind’. As the ‘engineers of orthopaedic surgery’ (Consultant Surgeon, C), Innovators tested the boundaries of EBP, as illustrated in the following quote:

“So, I came up with an idea. I was very clear in my own head that this was a definite innovation; it would definitely potentially make a difference.” (Consultant Surgeon, A)

Paragon surgeons interpreted ‘going beyond the evidence’ negatively. The claims of expertise emanating from Innovators were unacceptable to them because variation was generated through ‘risky’ uncontrolled innovation:

“I don't think you can convince these people that they're taking massive risks with people's quality of life, making changes [to practice] because they're innovators, [they believe] they're there to make things better. They have an unshaken belief, you know, it's a bit like a personality disorder.” (Trainee Surgeon, B)

Both Innovators and Paragons believed that clinical practice could be improved, but they differed in how this might be achieved. Innovators worked at the microlevel; they were attempting to improve practice—patient by patient. Paragons adopted a more macro‐level approach that envisioned change achieved across populations. For Paragons, evidence from large clinical trials, and improvements that could be tested and applied nationally, were critical:

“You might find small sub‐groups of patients where you want to push the envelope a little bit with one technique or another. But that should not be done by a lone surgeon in a lone department somewhere with no research support and no ability to follow up the results, no actual research question that they are asking in the first place. They are just tinkering and trying out a few things.” (Consultant Surgeon, A)

Maverick surgeons did not appear to prioritize innovation or improvements derived from research. They had the ‘fortitude’ to go against the status quo and test new ways of practising, they did not need to know how a procedure worked, they just had to judge for themselves that it did. We identified more Maverick types and behaviour in Case B than in A and C which signified the impact of place on practice. Surgeons in Case B appeared to be able to maintain a high level of autonomy and status, which enabled them to resist challenges within their organization. Maverick surgeons in particular displayed this behaviour:

“So, I got told I can't use drains for my replacements, okay? Which I found repugnant, because I was told by somebody who isn't a surgeon, I can't use a drain. I think that is awful because you're undermining the surgeon who's trying to do the operation. They [evidence producers] argue they make no difference, well, you know, you can look at the difference in my patients.” (Consultant Surgeon, B)

Mavericks in Case B became angry and protested when managers attempted to reduce the use of nonevidence‐based implants by removing them from the storage rooms and preventing new orders being placed. In one departmental meeting, a surgeon protested loudly that decisions were ‘made according to finances’ (Observation note, B). Mavericks mistrusted scientific evidence, and decisions made by nonorthopaedic professionals and they continued to use their favoured implants. Other surgeons working in Cases B and C queried how Maverick surgeons were ‘able to get away with it’ (Consultant Surgeon, B) but this latent disagreement often simply reaffirmed the Maverick identity rather than generated conflict within the speciality: one referred to a Maverick as ‘absolute cowboy’ (Consultant Surgeon, C).

Mavericks viewed standardized medical devices as dreary when compared to the latest technologies. We found evidence of commercial influence from manufacturer representatives across all three of our cases potentially acting as a factor in this enthusiasm for new technologies. For example, in Case A, company representatives gave out leaflets, in Case C, they would circulate in theatres providing advice. Mavericks in all cases were more inclined to favour ‘shiny new kit’ over standard supplies but unlike the Innovators, this was not because they represented technological advancements, but rather that this helped to enforce Maverick status. One Paragon surgeon described:

“So, I know some surgeons who are mavericks, who see a shiny new piece of kit and go, oh, I like that, I will have that, and I will use this. I'm going to use that and off they go.” (Consultant Surgeon, A)

The typology was functional, in that it aided our analysis and helped make sense of the empirical data within an emerging identity framework (i.e. to uncover the dimensions of identity described in Table 2) (McKinney, 1969). However, the typology was also explanatory. It pushed us to uncover why surgeons working in different places enact their varied identities to enhance legitimacy and stratify themselves in their response to EBP.

### Identity tactics to enhance legitimacy and stratification in responding to EBP

During the analysis, and using feedback from the hospital seminars, the typology was refined further by systematically searching the analytic dimensions of identity for meaningful relationships, empirical regularities and differences between surgeons working in different places (Kluge, 2000). For instance, we sought examples of what was ‘trusted’ by different surgeons and what was considered ‘legitimate’ knowledge in different settings. We returned to the literature and noted that McDonald and Harrison (2004) and others found that clinicians resisted guidelines (Grimshaw & Russell, 1993; Grove et al., 2018), although our analysis suggested that particular forms of resistance were more routinely associated with certain types of identity, for example, in Maverick surgeons in Case B with high organizational autonomy (they were ‘able to get away with it’ (Consultant Surgeon, B)).

We explored the impact clinical guidance had on routine work in each hospital and found stark differences which lent support to our emerging interpretations about why the types develop, and how organizational contexts might reinforce types. The professional identities appeared to relate to organizations or ways of organizing practice across different types of hospital. A Paragon described how in their university (teaching) hospital ‘the clinical trials stuff has been embedded in their [surgical trainees] training for as long as they can remember, it's normal. It is second nature’ (Consultant Surgeon, A). The way organizational practice was orientated towards, and rewarded research in Case A, appeared to mutually attract and reinforce surgeons and trainees who had a propensity towards EBP.

Surgeons and associated staff talked about themselves and other surgeons (real or imagined) as aligning to a particular identity…‘I'm the innovator ‐ that's what I do’ (Consultant Surgeon, A) as if types were bounded and consistent over time. However, our observational data revealed that individual surgeons did not always act consistently, and identities were sometimes blurred. Surgeon identities were not evenly distributed across our cases and the perceived value of the different types appeared to vary by place. There were more Paragons in Case A (university teaching hospital), whereas Mavericks appeared to dominate in Case B (general hospital). Innovators were spread throughout the cases, but with many instances in Case C. Innovators, were frowned upon by Paragon surgeons in Case A, the self‐described Innovator above was described as ‘rogue’ by a colleague. Innovators were more accepted in Cases B and C. Case C contained the most variation in types of surgeon, but even here surgeons tended to cluster around one type. Nonetheless the overarching narratives around EBP seemed to cluster around identity and place.

Revisiting the surgeon typology through our abductive analysis enabled us to structure and group the goal of surgeon's identity work (Timmermans & Tavory, 2012). This led us to reinterpret the data as a set of identities which were constructed for specific purposes; firstly, as rhetorical devices which signify surgeon's position as authentic and legitimate (Fox, 1992; Jeffcutt, 1994) and secondly, as a strategy to stratify themselves from colleagues (Pope, 2003; Powell & Davies, 2012; Timmons & Tanner, 2004). The identities appeared to support different responses to standardization, and could be used as a predictor of adherence to EBP. The different types were mobilized to resist competing narratives which might challenge beliefs regarding EBP (Harrison, 2002; Harrison & Smith, 2004), for example, dismissing guidelines as ‘stifling innovation’.

Unsurprisingly, given that it has dominated medical practice since the 1990s, the foundations of EBP were recognized and understood among the surgeons. They described ‘levels of evidence’ referring to canonical texts which outline the ‘five levels of evidence [which] should be assigned for all clinical articles’ (Marx et al., 2015) and they were aware of clinical guidelines. However, the enactment of EBP varied by type. Mavericks and Innovators opined that everyday orthopaedic work could not simply be directed by external evidence (McDonald & Harrison, 2004; Pope, 2003) and were keen to articulate their perceived difference, positions of power, status and authority compared to other professional groups (Fox, 1992; Grove et al., 2020). Such attempts to stratify themselves came into play often when there was a requirement to follow guidelines, or to conform to organizational rules generated by nonorthopaedic surgeons.

All types of surgeon argued that orthopaedic surgery could not be directly compared to other interventions in medicine. This created interprofessional boundaries which made claims to status or power differences (Powell & Davies, 2012). As one orthopaedic surgeon explained: ‘whereas a physician might sit back and think about a problem in the coffee room and deliberate, our job isn't like that. You know, it's immediacy, you need to have an immediate decision’ (Consultant Surgeon, A). The exercise of real‐time judgement and the need to respond to case and external contingencies echoes previous work, (Ducey & Nikoo, 2018; Pope, 2002), but identity work influenced how this was reconciled in practice.

We found instances of intraprofessional identity boundary setting (Powell & Davies, 2012). These within speciality differences appeared to reflect how surgeon types differentially placed themselves in the clinical hierarchy with reference to varied approaches they espouse towards EBP, for example, a Maverick surgeon described not following guidelines as a ‘tradition’ within orthopaedics:

“There are people who don't follow policy…evidence‐based guidance you know. I think it's an orthopaedic tradition, it's just more characteristic of us [orthopaedic surgeons].” (Trainee Surgeon, C)

Another Maverick surgeon mistrusted information that was not produced ‘by hip guys, you know…people like me’ (Consultant Surgeon, C). The privileging of technical orthopaedic knowledge by Mavericks and Innovators was used to retain power and status over their decisions and actions. As described by Fox (1992), these surgeons felt able to reject attempts to standardize their practice in favour of what they considered legitimate surgical knowledge (which could only be developed and defined by other orthopaedic surgeons): this is illustrated in this quote from an Innovator surgeon:

“There is very little sort of robust evidence to guide practice, so you rely on other surgeons' anecdotal experience and your normal practice to help guide what works and what doesn't in orthopaedics.” (Trainee Surgeon, B)

Mavericks routinely identified problems with scientific evidence, particularly the inappropriateness of randomized trials for their speciality. They argued that trial evidence was lacking, was of low quality, not needed or conducted on inappropriate patient groups. One Maverick suggested that:

“99% of what we do in orthopaedics is evidence free, most of what we do…is we do what we do… you can’t provide any evidence basis for it. It’s what’s been done and apparently seems to work.” (Consultant Surgeon, C)

Aside from technical disputes, Mavericks and Innovators negotiated the contingent nature of surgical work (Heath et al., 2018; Pope, 2002; Svensson et al., 2009) by referring to the ‘art’ and ‘craft’ of surgery. Externally mandated research evidence was incongruous: surgery was an act of ‘faith’ in what they could personally achieve, not a discipline amenable to standardized guidelines:

Your craft and experience will shape the way you believe something should be done. There's almost an element of faith about it. It's like we're all Christians, but there's some Catholics and some Protestants. We all know basically where it needs to be. But there will be those of us who feel strongly, actually I want to do it this way and others [who say] I want to do it that way. Nobody knows which is right. (Consultant Surgeon, A)

In contrast, Paragon surgeons saw such arguments as a myth mobilized by ‘rogue surgeons’ to justify their behaviour (Consultant Surgeon, A). One Paragon surgeon said:

“I don't think there's much art [or] artistry involved, it's based on evidence and if you get respected people to look at that evidence and develop policy, then that's really useful.” (Trainee Surgeon, B)

This stratification process signifies the power struggles within the orthopaedic speciality, where surgeon types compete for position. There were differences between Mavericks and Innovators. Maverick surgeons might dismiss guidance, by suggesting ‘NICE [guidance], is irrelevant. They don't tell me anything’ (Consultant Surgeon, C). Innovators were more ambivalent, unable to convert trial evidence for their own experiential practice because ‘their’ patients did not resemble patients in clinical trials. Innovators relied on personal experience of an intervention prior to it becoming part of their practice. Observations of surgical planning meetings in Case C, uncovered a perceived technical specialism possessed by Innovators who made claims of going beyond the evidence to test the boundaries of the ‘gold standard’ in surgery. This technical specialism sought to enhance the position of Innovators when compared to their colleagues on the ‘surgical treadmill’ (Consultant Surgeon, C). These discussions involved complex ‘high‐end’ surgery, that your ‘average hip surgeon wouldn't do’ (Observation note, C). These Innovators seemed to especially enjoy describing the highly technicality demands of cases where 3D printed implants were used and traditional standardized hip implants were perceived as inferior options compared to the customized ‘patient‐specific’ and ‘personalized’ implants that they used (Observation note, C).

Paragon surgeons repeatedly performed the same types of surgery using established, highly rated (i.e. evidence based) implants. One Paragon surgeon said he: ‘operates within a fairly limited framework of prostheses (devices), all of which are 10A [highly] rated. I don't do any experimental procedures on patients’ (Consultant Surgeon, B). His theatre nurse described his practice as so predictable that he could ‘set his watch by him’ (Orthopaedic Nurse, B). For these surgeons, standardization was viewed positively, making their work ‘run of the mill’ (Consultant Surgeon, A) and allowing a ‘production line’ approach (Consultant Surgeon, A):

“We [orthopaedic surgeons] are fortunate in that we have got operations that work well with large treatment effects. So, you don't need to tinker with it … patients are going to do very well. So yes, just deliver the service as simply and safely and as reproductively as you can, which is where following protocols is best.” (Consultant Surgeon, A)

The surgeons and staff we interviewed talked about themselves, and other surgeons as aligning or adopting a style of practice with seeming consistency. However, our observations revealed that individual surgeons did not always act consistently within a single identity, for example, surgeons could be mostly Innovators with occasional Maverick moments. This, at times could be influenced by organizational contingencies and features of place: as one prominent Innovator complained: ‘[patients] all get the same thing when the [surgical] list is long’ (Consultant Surgeon, C) indicating that innovation could be limited in the face of hospital pressure to increase the number of operations performed. We also found that Maverick behaviour could be partially curtailed in Case C once nonevidence‐based implants were removed from storage rooms (Observation note, C).

The surgeons’ identity work through which prestige and power were gained echoes Pope (2002) and Fox's (1992) findings, where professionals stratified themselves from others through identity tactics to elevate their position. We found that within the orthopaedic speciality, surgeons appeared to stratify themselves from others through a desire to cement their place in the clinical hierarchy. The three surgeon identities we identified seemed to draw on identity tactics and different discourses to enhance legitimacy both within and outside of the profession and justify their differing approaches to EBP.

## DISCUSSION

Our findings demonstrate that variation continues to be normatively supported in the context of orthopaedic surgical work. We show that variation can be understood and justified as a consequence of how surgeons adopt and enact distinct professional identities which serve a purpose to their practice: as a rhetorical device to signify position as authentic and legitimate and as a strategy to stratify themselves from colleagues relative to each other in responding to EBP. The different types were mobilized to resist competing narratives which might challenge beliefs regarding EBP (Harrison, 2002; Harrison & Smith, 2004), for example, dismissing guidelines as ‘stifling innovation’. The idea that identities appeared to support different responses to standardization, suggests that the typology could potentially be used as a predictor of adherence to EBP.

We argue that professional identity types and responses to EBP cluster in ways that serve to legitimize and reinforce surgeons’ approach to surgical work in the face of standardization (Timmermans & Berg, 1997). Fox described how the tacit nature of medical knowledge allows surgeons to preserve their identity as autonomous, self‐regulating professionals (Fox, 1992). The bolstering of surgical identity occurs through the cyclical social process of legitimizing the act of surgery and the power this discrete knowledge and action generates, which in turn reproduces legitimation (Fox, 1992). We found that intraprofessional identity work goes beyond the power surgeons manifest to legitimize their practice to outsiders, and also works to generate power claims and hierarchies within the orthopaedic profession. Building on Powell and Davies (2012), we saw how surgeons engaged in different forms of identity work which enabled them to rationalize (non)adherence to standardization compared to others. We saw varying dimensions of identity work (autonomy and discretion, client interest, knowledge jurisdiction) which surgeons used differently to negotiate stratification of themselves within their own discipline. Therefore, the surgeons actively sought to differentiate themselves from their intraprofessional colleagues.

### Surgeon identity and orientation to EBP

We extend the work of McDonald et al. (2006) on identity narratives to show that failure to follow EBP was associated with the identities individuals create and negotiate, and with narratives used to legitimize differing responses to EBP. Moreover, surgeons' propensity to follow EPB was not only related to identity but also influenced by place. Identity types were not evenly distributed across the different organizational settings. As might be expected, there appeared to be more Paragon surgeons in Case A (academic university department) (Damschroder et al., 2009). Mavericks dominated Case B (general hospital) and Innovator types were distributed across the cases, but with many examples in Case C, the teaching hospital. This suggests that microlevel differences between surgeons (i.e. training) do not entirely explain practice variation, and that there are structural factors at play in creating or maintaining types. Through our case selection, we anticipated an organizational analysis which focused on hospitals' organizational structures and the formal processes of guideline implementation (Grove et al., 2015). However, we discovered a more microlevel understanding to explain why practice cannot be easily standardized finding professional specification within a clinical tribe. We were able to show how we need to go further than an organizational analysis, into the clinical tribe, to see variation in how surgeons enact identity in their orientation to EBP to stratify themselves inter‐ and intraprofessionally.

These findings extend Pope's (2002) work on surgeon contingencies to show how individual surgeon identity work takes place within the professional community to shape practice variation. The clustering of more Mavericks and Maverick‐type behaviour in one case study (Case B) appeared to be a direct consequence of the high autonomy and power surgeons held in this hospital. This allowed Maverick surgeons to resist external challenges such as requests to standardize implants. Only one of the cases, Case A, was consistently positive towards research evidence, and we posit this as unsurprising, given its affiliation to a University geared to creating such evidence.

We found some evidence to suggest that hospitals seemed to house different identify types. However, insight would be gained from future organizational and structural exploration to discover whether a surgeon would change their type if they went to another institution, or if the organizations and surgeons select each other to fit ‘innate’ types. How positioning as a type is embedded and established is also important—for example, is it at medical school or in the early years of training as a surgeon? The formation of medical professional identities has been described through a process of professional socialization, where work norms and values, and knowledge and skills adapt the beginner to the culture of the experts (Cruess et al., 2014, 2015). In practice, the socialization of medical professionals is influenced by the health‐care system and organizational environment (Cruess et al., 2014). Therefore, medical professionals may adapt further to become members of an organization, and are aware of belonging to the hospital community (Lave & Wenger, 1999).

### Professional identity for status and stratification

The typology offers new insight into the relationship between surgeon identity and the bureaucratization of medicine, through policy‐mandated standardization of surgical practice (Harrison, 2002). We have used the typology to extend and contribute to the literature on identity theory (Stets & Burke, 2000) and discussed how surgical variation is generated through distinct characteristic types which evolve for a specific purpose (McDonald et al., 2006). The typology has allowed us to analyse how surgical decisions and actions were understood and legitimized, to see how surgeons used professional identity tactics as rhetorical devices, and to signify position and status in relation to others, to established norms and to national guidelines. How surgeons responded to practice uncertainty, for example, the introduction of a new clinical guideline, appeared to be shaped by the identity work of surgeons. Surgeon type helped us to understand uncertainty, particularly for Innovators and Mavericks who appeared to struggle to rationalize uncertainty in their work (Mavericks resisted requests to use standardize implants they have not previously used). Paragons appeared able to acknowledge uncertainty. They understood that at times uncertainty and clinical equipoise exist, and it is through evidence and research that uncertainty can be responded to. Across all the cases, we observed surgeons using their ‘type’ (and the components of their identity work) to defend and reinforce their position, actions or decisions.

Our analysis extends the work by Fox (1992) and shows the ways in which the differences in professional identity are enacted by orthopaedic surgeons. The identity types allow surgeons to define their own practice, to protect their own work boundaries in relation to outsiders and surgical insiders and contest EBP. Surgical identities were used to promote the status of orthopaedic surgery above others in the hierarchy of clinical and managerial specialities (Powell & Davies, 2012; Stets & Burke, 2000), and as a device for intraprofessional stratification between orthopaedic surgeons. In our typology, the three types of surgeon made status claims to position themselves, and their knowledge as powerful. The surgeons in our study made status claims that they are ‘better’ (Paragon) because they follow research evidence, or because they take a more nuanced view of evidence (Maverick) or because they are able to go beyond the evidence (Innovators). Thus, surgeons used their identities to defend their status, and to legitimize their practice.

We suggest that this typology can be used to predict surgeon adherence to EBP. Innovators were more likely to reject EBP because it was not experientially anchored in their ‘heads or hands’. Mavericks were often indifferent to research evidence and guidelines and, therefore, resisted standardization. Pope (2002) noted that ‘surgeons have a vested interest in presenting their practices as skilled and complex and their work as contingent’ (p. 381). Surgeons may have similar vested interests in the identities presented here. By exploring the typological differences across different case settings, we have been able to tentatively explore how this ‘presentation of practice identity’ plays out differently to generate variation. Adherence to EBP appeared to be patterned by place: in effect surgeons collectively developed ontological sanctuary thus legitimizing their different behaviours in response to external attempts to standardize their practice.

### Implications of these findings on health‐care practice

Variation in the practice of hip replacement will persist while the surgeon types remain unchallenged. Our respondents recognized surgical variation as idiosyncratic, but we have suggested that there is patterning and purpose to this variation. We are not in a position to judge issues of surgical safety but there are some important moral and social consequences and potential longer term negative effects of some of the responses to EBP we have described (e.g. there are financial implications associated with Maverick surgeons selecting expensive devices). Similarly, there are potential risks to patients receiving interventions trialled by Innovators, which may not have been proven effective or safe in clinical trials.

This moral dimension of professional identity work was not central to our data and analysis but came to light in our reflections about what the typology and identity tactics meant for patients and those accessing health services. Health‐care policy continues to pursue EBP and standardization as a priority (McDonald et al., 2006; Wilhelm et al., 2020) but surgical variation persists. Patients scheduled for surgery do not know which type of surgeon will perform their surgery and may not be aware of this variation. In addition, we have suggested that different hospitals may potentially attract or support particular surgical identities and this means that organizational change may be needed to make significant impact on practice variation. Future research would need to investigate the stability of the types across organizational settings or ways of organizing to determine how innate or organizationally dependent the types are. There is scope to explore the moral dimensions of surgeon identity work, and how change could be achieved at both the micro and organizational levels.

Our study has some limitations; notably the small number of cases we studied. It is possible that increasing the sample size would increase the number of types identified or the overall typology, however, we have confidence in the types we have found. The three hospitals in our study were located in major towns or cities and practice may differ in rural contexts or outside the United Kingdom. We also did not consider the influence of private practice on our typology, although we have some limited observations from professional meetings and conferences that suggest that the private sector may encourage Maverick behaviour and provide a ‘test bed’ for Innovators. Elsewhere, Waring and Bishop (2012) have described how commercial pressures shape service delivery when surgeons take on more private work and this is an area where our typology could be further tested.

## CONCLUSION

Our study has shown the ways in which identity types are used by the surgeons and others to legitimize their practice, decisions and relationship response to EBP. We found that intraprofessional identity work goes beyond the power surgeons manifest to legitimize their practice to others. The three characteristic identities presented in our study, Mavericks, Innovators and Paragons, present differing accounts of how surgeons claim legitimacy over their work to preserve their interests and to maintain their autonomy and status in the clinical hierarchy. These identity types serve a purpose and provide the ‘backbone’ to a surgeon's practice—influencing their talk, action and interaction with others. Surgeon identity is used as a rhetorical device to signify position and as a strategy to stratify the profession. In addition, it appears to be a predictor of adherence to EBP.

We argue that surgeon type can predict and explain variation in how surgeons respond to standardization and in doing so we contribute new insight and understanding into the relationship between surgeon identity and the bureaucratization of medicine. Our typology of surgeon identity extends and contributes to the literature on identity theory, to show how surgical variation is generated through distinct characteristic types which evolve for a specific purpose. We found surgeon identity types occur on a continuum, but they cluster around the three types we have identified.

The typology we have presented here is not only a matter of character, despite the colourful names we have given to the types. Being operated on by a Maverick, an Innovator or a Paragon influences the surgery a patient receives. We found surgeon identities also clustered in place, therefore, there may be an organizational element to reinforcing certain types, or the altering surgeon's position on the identity continuum. If this is the case, then surgical variation will not be fully addressed through approaches that focus on training the individual surgeon on EBP or mandating individual adherence to guidelines. Further research attending to organizational settings is important. We found that surgeon identity work takes place within the professional community to shape practice variation. Therefore, we need to understand the power dynamics between surgeon types and the flexibility and transferability of inter‐ and intraprofessional identity work which occurs to support EBP.

## CONFLICT OF INTEREST

All authors declare no competing interests with regard to the submitted work.

## AUTHOR CONTRIBUTIONS

**Amy Grove:** Conceptualization (lead); Data curation (lead); Formal analysis (lead); Funding acquisition (lead); Investigation (lead); Methodology (lead); Project administration (lead); Validation (lead); Writing‐original draft (lead); Writing‐review & editing (lead). **Catherine Pope:** Formal analysis (supporting); Methodology (supporting); Supervision (supporting); Validation (supporting); Writing‐original draft (supporting); Writing‐review & editing (supporting). **Graeme Currie:** Conceptualization (supporting); Data curation (supporting); Formal analysis (supporting); Funding acquisition (supporting); Investigation (supporting); Methodology (supporting); Project administration (supporting); Supervision (supporting); Validation (supporting); Writing‐original draft (supporting); Writing‐review & editing (supporting). **Aileen Clarke:** Conceptualization (supporting); Formal analysis (supporting); Funding acquisition (supporting); Methodology (supporting); Supervision (equal); Validation (supporting); Writing‐original draft (supporting); Writing‐review & editing (supporting).

## ETHICAL APPROVAL

The study was approved by the University of Warwick Biomedical and Scientific research Ethics Committee, England (approved 2 June, 2014; reference number REGO‐2014–645) and the Research and Development departments of each of the three hospital sites (Case A approved on 30 June 2014, Case B approved on 23 October 2014, Case C approved on 21 August 2014).

## PATIENT CONSENT STATEMENT

In line with the ethical approval, each participant signed an informed consent form and were provided with information outlining the purpose of the study and their rights to withdraw. Informed consent was obtained to include anonymized data in any publications. Confidentiality is protected as the data are anonymized.

## PERMISSION TO REPRODUCE MATERIAL FROM OTHER SOURCES

The manuscript contains no material from other sources.

## Data Availability

The data sets generated and analysed during the current study are not publicly available due to restrictions of ethical approvals obtained for this study.
